# Ongoing Evolution in the Genus *Crocus*: Diversity of Flowering Strategies on the Way to Hysteranthy

**DOI:** 10.3390/plants10030477

**Published:** 2021-03-03

**Authors:** Teresa Pastor-Férriz, Marcelino De-los-Mozos-Pascual, Begoña Renau-Morata, Sergio G. Nebauer, Enrique Sanchis, Matteo Busconi, José-Antonio Fernández, Rina Kamenetsky, Rosa V. Molina

**Affiliations:** 1 Departamento de Gestión y Conservación de Recursos Fitogenéticos, Centro de Investigación Agroforestal de Albadaledejito, 16194 Cuenca, Spain; maria2.1984@yahoo.es (T.P.-F.); mde@jccm.es (M.D.-l.-M.-P.); 2 Departamento de Producción Vegetal, Universitat Politècnica de València, 46022 Valencia, Spain; begonya.renau@uv.es (B.R.-M.); sergonne@bvg.upv.es (S.G.N.); esanchdu@bvg.upv.es (E.S.); 3 Department of Sustainable Crop Production, Università Cattolica del Sacro Cuore, 29122 Piacenza, Italy; Matteo.Busconi@unicatt.it; 4 IDR-Biotechnology and Natural Resources, Universidad de Castilla-La Mancha, 02071 Albacete, Spain; JoseAntonio.FPerez@uclm.es; 5 Department of Ornamental Horticulture and Biotechnology, The Volcani Center, ARO, Rishon LeZion 7505101, Israel; vhrkamen@volcani.agri.gov.il

**Keywords:** biodiversity, synanthous, hysteranthous, flower development, flowering time, genetic variability

## Abstract

Species of the genus *Crocus* are found over a wide range of climatic areas. In natural habitats, these geophytes diverge in the flowering strategies. This variability was assessed by analyzing the flowering traits of the Spanish collection of wild crocuses, preserved in the Bank of Plant Germplasm of Cuenca. Plants of the seven Spanish species were analyzed both in their natural environments (58 native populations) and in common garden experiments (112 accessions). Differences among species observed in the native habitats were maintained under uniform environmental conditions, suggesting a genetic basis for flowering mechanisms. Two eco-morphological types, autumn- and spring-flowering species, share similar patterns of floral induction and differentiation period in summer. The optimal temperature for this process was 23 °C for both types. Unlike Irano-Turanian crocuses, spring-flowering Spanish species do not require low winter temperatures for flower elongation. Hysteranthous crocuses flower in autumn prior to leaf elongation. We conclude that the variability in flowering traits in crocuses is related to the genetic and environmental regulation of flower primordia differentiation and elongation prior to emergence above the soil surface. The elucidation of the physiological differences between eco-morphological types of crocuses: synanthous with cold requirements and synanthous and hysteranthous without cold requirements, unlocks a new approach to the flowering evolution of geophytes in Mediterranean regions. *Crocus* species can serve both as a new model in the study of the molecular basis of hysteranthy and for the purposes of developing the molecular markers for desirable flowering traits.

## 1. Introduction

The diversification of plant life strategies is a keystone in the evolution and distribution of species in new environments. In nature, plants develop various chronological sequences of leafing, flowering, and dormancy in order to adapt to climatic conditions and to take advantage of pollination, nutrition, or ecological interactions. One example of a successful life strategy is the hysteranthous floral rhythm, which may be considered as a character of phylogenetically advanced forms in different plant groups, especially in the geophytes and deciduous trees [1,2]. As opposed to the synanthous species, in which leaves and flowers occur in the same season, hysteranthous plants produce vegetative and reproductive organs in different seasons. Since the photosynthesis rate in hysteranthous plants is low from flower emergence until anthesis, flower stem elongation and flowering occur on account of storage reserves. Hysteranthous trees are found in *Rosaceae, Calycanthaceae, Magnoliaceae,* and *Oleacea* families. Early flowering prior to leaf unfolding ensures that these trees pollinate, since abundant flowers attract more insects. These plants invest food reserves in reproduction before other organs begin to grow, while the rest of the growing season is dedicated to growth and storing reserves for the winter. In hysteranthous trees, both vegetative and reproductive organs require cold for dormancy release, but flower buds have lower heat requirements in comparison with leaves [2].

Another large group, hysteranthous geophytes, flower in a leafless stage during autumn in the areas with a Mediterranean climate [1]. Classic ecological research [3,4] suggested two main evolutionary pathways of hysteranthy in the geophytes: (1) In the *Urginea* type (*Urginea, Scilla, Narcissus*, and *Pancratium*), flowering is delayed to the next favorable season and seed dispersal and germination occur immediately after flowering; (2) in the *Crocus* type (*Crocus, Colchicum, Merendera,* and *Sternbergia*), flowering is advanced to the beginning of the new reproductive cycle, but seed dispersal and germination are delayed to the spring and following autumn [4]. Shmida [5] assumed that the plants of the *Crocus* type originated under “arid-alpine” conditions, with extremely dry summers and hard winters, and spread into Africa and the Iberian Peninsula from the high mountains of the Middle East.

Intermediate forms between synanthous and hysteranthous species in the same genus can be found in different habitats in the Mediterranean areas. For instance, *Scilla autumnalis* is a sub-hysteranthous or hysteranthous [3], while *S. bifolia* blooms late in winter or spring as a synanthous plant [6,7].

A full range of synanthous and hysteranthous species and their intermediate forms can be found between around 100 species of the genus *Crocus* (*Iridaceae*). The genus is widely distributed in the old World from Morocco and Portugal to western China. Turkey is considered the center of the variability of crocuses [8], while most species are found in the Mediterranean areas. Autumn hysteranthous and sub-hysteranthous crocuses flower from October until December, and spring synanthous species flower from February until May [8,9,10,11]. Many spring crocuses are popular ornamentals [12], but the most important species is the autumn-flowering saffron *C. sativus* L., one of the world’s most expensive spices [13,14]. Other species are also of potential pharmaceutical interest [13].

Similar to other geophytes, crocuses survive the unfavorable seasons not only by means of seeds, but also through specialized underground storage organs, tunicated corms, with renewal buds resting underground. The flower of the crocuses is epigynous, with an inferior, subterranean ovary borne upon a scape (stalk). The perianth rises from the top of the ovary in the form of a long tube, which serves instead of a stem to carry the showy part of the flower above the surface. After fruit set, the scape elongation leads to the capsule emergence. Thereafter, fruit ripening takes place [9].

Until now, physiological studies of the reproductive phenology of *Crocus* have focused on the cultivated saffron [14,15,16,17,18] and on several spring crocuses, which are popular in the flower industry [12]. In 2007, the European Commission and the Spain and Castilla-La Mancha Governments funded the *World Saffron and Crocus Collection* (WSCC), one of the most extensive germplasm collections in the world [19]. The purpose behind the creation of this collection was to slow down the intense genetic erosion of the saffron crop over the last few decades and boost the preservation of a wide variety of crocus genotypes, which are potential carriers of useful genes for crop improvement, e.g., resistance to biotic or abiotic stresses, biosynthesis of secondary metabolites, etc. At present, the germplasm collection is maintained at the Bank of Plant Germplasm of Cuenca (FAO code ESP124) and comprises about 350 accessions of 62 species (excluding saffron accessions), 128 of them belonging to seven wild Spanish species. Both autumn and spring crocuses are well represented in the Spanish collection, thus enabling an in-depth analysis of their intraspecific and interspecific variability and an understanding of the evolution of flowering biology in crocus species with various seasonal patterns, from synanthous to sub-hysteranthous and hysteranthous.

In this report, we provide a detailed analysis of the phenological variability within and among the different species of wild crocuses on the Iberian Peninsula with diverse life strategies and analyze the effect of the temperature on the flowering processes in these species.

## 2. Materials and Methods

### 2.1. Flowering of Spanish Crocuses in Their Natural Environment

Flowering time and distribution of Spanish crocuses (*C. serotinus, C. nudiflorus, C. clusii, C. cambessedesii, C. nevadensis, C. carpetanus, C. vernus)* in natural populations was analysed using passport information of 58 accessions of the seven wild species from the Spanish germplasm collection, located in the Agroforestry Research Centre of Albdalejito (CIAF-IRIAF) in Cuenca (Castilla-La Mancha, Spain). The data related to each species, accession number, latitude, longitude, and elevation of the diverse collecting places are provided in Table S1. Detailed information on the accessions is maintained in the Documentation System of the BGV of Crocus [19]. Based on passport information, we calculated number of days from 1 August 2011 until flowering (DTF) for each accession and correlation between the beginning of flowering and the population altitude above sea level. The date of 1 August 2011 was selected for further comparison with plant development under controlled conditions.

### 2.2. Phenology of Spanish Crocuses Grown Ex Situ

The phenology of 112 accessions of seven species (Table S2) from the collection of the germplasm bank of CIAF-IRIAF was recorded during their annual cycle. The germplasm bank is located in the latitude and longitude 40°04′08.17″ N and 2°11′057.07″ W, respectively, at an altitude of ca. 1000 m.a.s.l. General conservation and multiplication strategies for the collection are standard for international genebanks, as outlined by Engels and Visser [20].

In the common garden experiment, corms harvested from the field at the end of June 2011, were stored at 23–25 °C until the end of July. On 1 August 2011, 20 plants of each accession were planted in two 12 L containers (10 plants per container) with planting mixture COMPO SANA^®^ Universal and sand (1:3). Planting depth was about 10 cm. Plants were grown in a net house under a polycarbonate cover. A drip irrigation system was used when necessary. Variations in temperature throughout the year are provided in Figure S1.

For each accession, we recorded the number of days after planting (DAP) when 50% of the population reached the following stages: (1) sprouting, when cataphylls are breaking through the soil surface; (2) leaf emergence or unfolding from cataphylls; (3) flowering; (4) capsule emergence and fruit ripening; and (5) leaf senescence.

### 2.3. Temperature Requirements for Flower Initiation and Differentiation during Corm Storage

The effect of temperature regime on flower initiation was studied on four species: autumn-flowering sub-hysteranthous *C. serotinus* BCU002775, autumn-flowering hysteranthous *C. nudiflorus* BCU002944, a late winter-early spring synanthous *C. nevadensis* BCU002695, and a mid-spring synanthous *C. vernus* BCU002998 (Table S2). After harvest at the end of June 2011, 40–50 dry corms of each species were stored at constant temperatures of 10 °C, 23 °C, and 30 °C, and a relative humidity of 60–75%. Apical meristems of four to five corms of each species were monitored with an Olympus Stereomicroscope System SZ61 at regular intervals (5–7 days) to record the differentiation stages of the reproductive organs. Photographs were taken with a Nikon DS-Fi2 digital camera. The sampling of the apical buds began in mid-July and was completed at the time when most plants clearly showed floral differentiation in early September.

The experiment was repeated in the next season. In addition, *C. serotinus*, which differentiates floral primordia at 30 °C, was tested at 35 °C in this second year to find the upper inhibitory limit. Similarly, the storage of *C. nudiflorus* at 5 °C was tested to find its lower inhibitory limit.

### 2.4. Effect of Growth Temperatures on Flowering Time

An autumn species *C. serotinus* and two spring species (a late winter *C. nevadensis* and a mid-spring *C. vernus*) were used in this experiment (Table 1).

After harvest at the end of June, dry corms were stored at 23 °C and then planted in mid-September 2010 in 12 L containers with planting mixture COMPO SANA ^®^ Universal and sand (3:1) and grown at three temperature regimes of constant 10 °C, 17 °C, and 25 °C (autumn crocus), or at two temperature regimes of constant 10 °C and 25 °C (spring crocuses) (Table 1). Regular irrigation with 50% Hoagland nº2 solution was performed [21]. Plants were maintained in growth chambers with lighting at 45 cm above the trays by means of two fluorescent cold white lights (CFL 13W 2700K Sylvania 4), and a photoperiod of 16/8 h (light/dark). Irrigation was provided to obtain a uniform moisture.

In addition, in order to elucidate an effect of low winter temperatures on flowering of spring species, we exposed planted corms to the sequence of 5, 2, and 5 °C from 20 November 2010 to 20 January 2011. From the second half of September to 20 November, the temperature gradually dropped to 5 °C (Table 1, treatments 6, 7). These conditions simulate the winter chilling period in natural habitats. After chilling, plants were grown at two constant temperatures, 10 °C and 17 °C. For each treatment, two to three replicates (autumn crocuses) or three to four replicates (spring crocuses) of 10–30 corms were used. During the chilling period, we recorded the length of the different parts of the underground growing buds of *C. nevadensis* and *C. vernus*.

Five plants per treatment were measured four times over a period of 80–100 days before blooming. At blooming, the average length from the soil of 10–15 flowers of both spring and autumn crocuses, the days when the corms are in bloom (flowering period), and the average number of days until these flowers begin to wilt (flower life), were recorded in each treatment, and the cumulative flowering curves were obtained.

### 2.5. Statistical Analysis

The data were compared and analysed by one-way ANOVA (Statgraphics Plus 5.1 for Windows, Statistical Graphics Corp.). Mean separations were carried out using the LSD multiple range test when the number of samples is low. When more than four samples were compared, the Scheffé’s method for multiple comparisons was employed. Generalized Linear Models (GLM) and Polynomial regression of the same statistical package were used to analyse the effect of the altitude on the flowering time in natural populations. The arcsine transformation of percentage data sets prior to analysis of variance was carried out.

## 3. Results and Discussion

### 3.1. Phenology and Flowering Patterns in Natural Habitats

**Figure 1:**
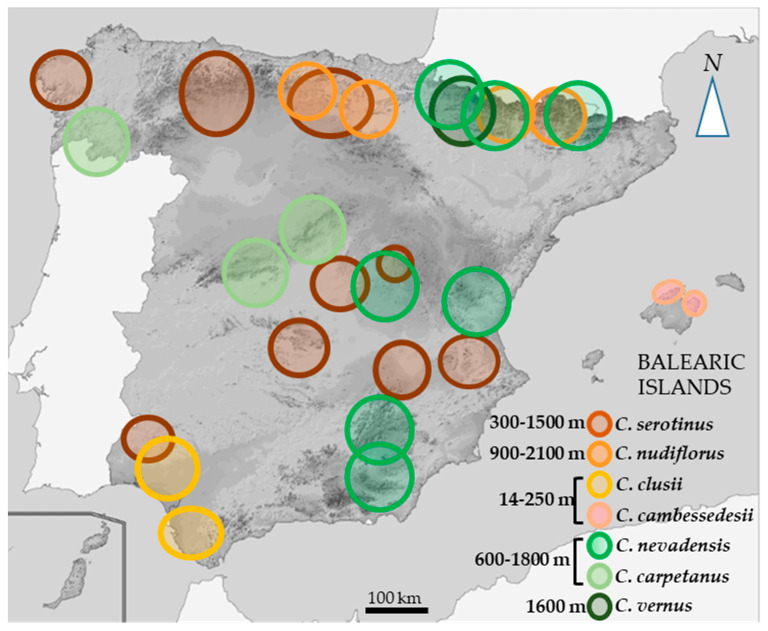
Areas of collection and altitude of distribution of seven wild *Crocus* species in Spain, as presented in the Documentation System of the BGV of Crocus [19].

An analysis of the passport data of seven studied species shows their wide distribution across Spain (Figure 1). Phenologically, two main groups of autumn-flowering and spring-flowering species are clearly distinguished (Table 2). Although in most cases species have distinct areas, occasionally autumn and spring species share the same habitat, e.g., *C. nevadensis* and *C. nudiflorus* or *C. nudiflorus* and *C. vernus* (Figure 1).

*C. serotinus* and *C. nevadensis* are the most widespread species, their populations being found in the north, east, and center of Spain. Both species are endemic to the Iberian-Maghreb region. *C. clusii* is closely related to *C. serotinus* and flowers in the same period, but it is confined to the coastal areas of southern Spain and Portugal. *C. carpetanus* is the nearest ally of *C. nevadensis* and also flowers in the same period; however, the distribution of the two species is different, *C. carpetanus* only being located in the Spanish Central System and Cantabrian mountains [18,21]. Both *C. clussi* and *C. carpetanus* are endemic to the Iberian Peninsula. *C. cambessedesii* is endemic to the Balearic Islands. *C. vernus* subs. *albiflorus* is found only in the Pyrenees, at 1300–2200 m a.s.l. [22].

Most Spanish crocuses are found in forested areas, although some populations of autumn-flowering crocuses can be found in coastal areas (in pinewood clearings).

Autumn-flowering species *C. serotinus* and *C. nudiflorus*, found mostly at 600–2000 m.a.s.l., flower from mid-September to the end of October. *C. cambessedesii* and *C. clusii* found at a lower altitude (14–250 m.a.s.l.; Table 2), flower in early November. A non-linear relation (R^2^ = 0.73) between flowering time and altitude was observed in autumn crocuses (Figure S2). In addition to the altitude, which largely determines temperature regime, water availability influences the time of sprouting and flowering. The locations of spring crocuses vary from 600 to 1600 m.a.s.l. and no correlation between flowering time and population location was found (*p* > 0.05). Two spring-flowering species, *C. nevadensis* and *C. carpetanus*, bloom at the very end of February, but some populations of *C. nevadensis* flower even earlier (around 16th February). *C. vernus* is the species that flowers the latest, blooming from late April to early May (Table 2).

Since populations of the same species can be found under very different environmental conditions [9,18,22,23], their flowering dates vary greatly. Both genetic and environmental variations affect these differences. However, the fact that spring and autumn crocuses sometimes share the same habitat points to significant inter-species genetic differences (Table 2).

**Figure 2:**
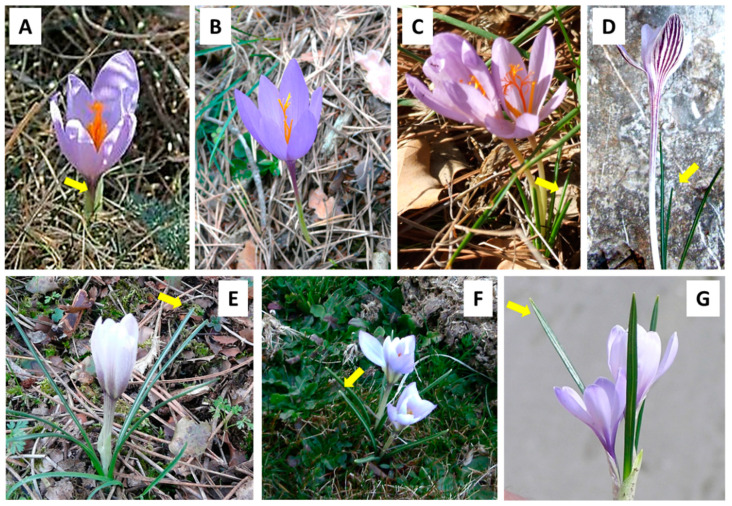
Leaf development (arrows) in flowering time of autumn-flowering *C. serotinus* (**A**); *C. nudiflorus* (**B**); *C. clusii* (**C**); *C. cambessedesii* (**D**) and spring-flowering *C. nevadensis* (**E**); *C. carpetanus* (**F**); *C. vernus* (**G**).

Most autumn-flowering species are synanthous or sub-hysteranthous: their leaves elongate shortly before, simultaneously with, or a few days after flowering (Figure 2). Only one species, *C. nudiflorus* (Figure 2B), is truly hysteranthous, and its leaves emerge several months after flowering. However, all spring-flowering species are synanthous. It should be noted that in native populations, cataphyll emergence only occurs in autumn species when the mean air temperature falls below 20 °C, and in spring species when the temperature rises above 4 °C (Table 2).

No evident relationship between flowering pattern and the temperature conditions of the location was recorded. On the contrary, spring and autumn species are very often found in the same environment. For instance, *C. nevadensis* cohabits with *C. serotinus* in the area of “Sierra de Cazorla” in Jaén [24], and *C. vernus* and *C. nudiflorus* grow in the same place in the Pyrenees, in Jaca (Huesca). However, two species never flower simultaneously in the same location.

The differentiation of flowering niches could be explained by the action of selective forces imposed by the environmental conditions, mutualists (pollinators and seed dispersal) or antagonists (floral pathogens and pre-dispersal seed predators). The selective forces imposed by mutualists seem to be the origin of the autumn crocuses’ life strategy. These species have larger, taller flowers than spring crocuses [18], a phenomenon described as “discovery advertisement” [25]. Plants from the Mediterranean region mainly flower in spring, and autumn flowering, as in the case of the autumn Crocus species, may be advantageous for pollination. The few simultaneously flowering species and the small number of pollinators may lead to a high degree of “forced” pollinator constancy and to a low rate of improper pollen transfer [26]. However, to date, knowledge of crocus pollination is limited. In turn, the survival strategy of the spring ephemeral crocuses is based on their flowering shortly after snow melt (as temperatures start to rise), reaching the complete development of their leaves before a full overstory canopy appears [27]. These species take advantage of the low-temperature regime and available moisture during this short growth period [28].

### 3.2. Phenology and Flowering in Common Garden Experiments Ex Situ

**Figure 3:**
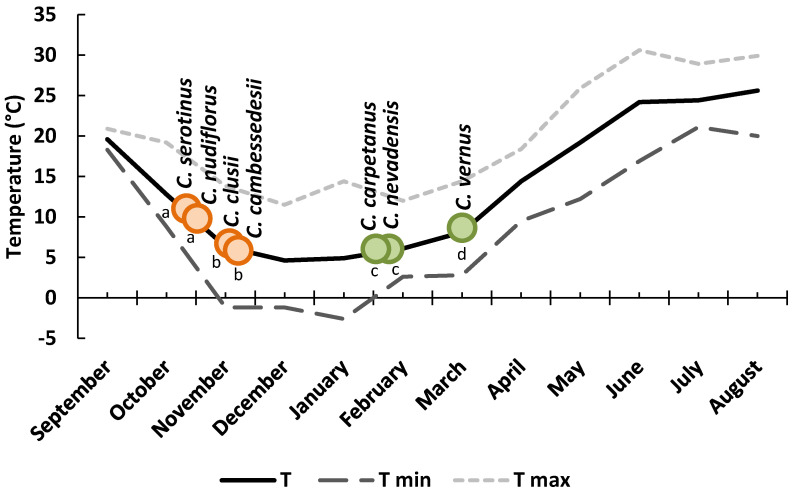
Flowering time of 112 accessions of seven crocus species under the same environmental conditions at the Bank of Plant Germplasm of Cuenca. Different letters indicate significant differences (*p* < 0.05) among species.

In order to assess the genetic and environmental components in flowering regulation, 112 accessions of the seven studied species were cultivated in the common garden experiment at the Bank of Plant Germplasm of Cuenca, and their phenological stages were recorded. In general, all of the species maintained the same phenological differences as in their natural environment (Figure 3).

**Figure 4:**
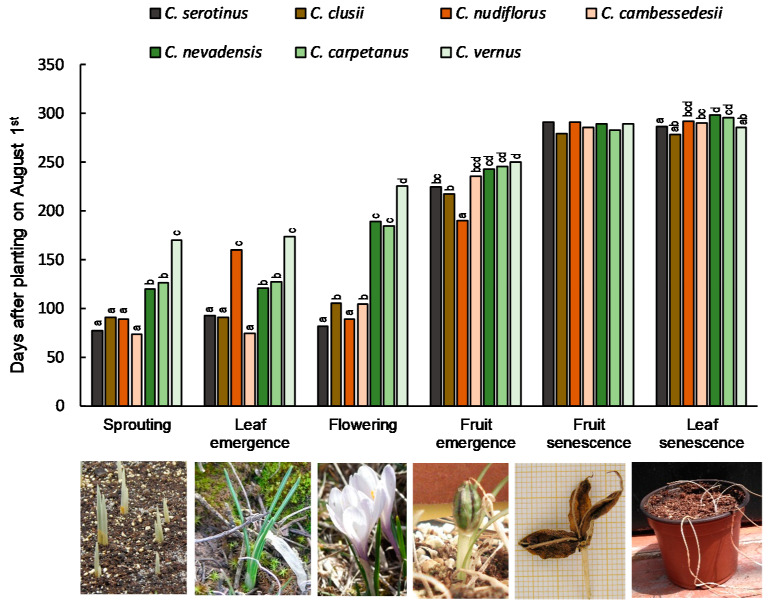
Differences in the phenology of *Crocus* species grown ex situ. One-hundred-and-twelve accessions of seven species from the collection of the *BGV* were assayed (Table S2). For each species, the mean value among the accessions of the number of days from 1 August (planting date) until 50% of the plants reach the phenological stage is represented. Twenty plants of each accession were measured. Different letters indicate significant differences (*p* < 0.05) among species.

In autumn-flowering species, leaf sprouting, unfolding, and growth took place shortly after (*C. serotinus*) or just before flowering (*C. clusii* and *C. cambessedesii*). However, in *C. nudiflorus*, the leaf sprouting occurred more than 2 months after flowering, showing a true hysteranthous pattern, as observed in the natural habitats (Figure 2 and Figure 4). As in the original environment, the earliest flowering (21–29 October) was observed in *C. serotinus* and *C. nudiflorus*, while *C. clusii* and *C. cambessedesii* flowered 15–24 days later (Figure 3 and Figure 4, Table 2).

In spring-flowering species, the cataphylls and leaves of *C. nevadensis* and *C. carpetanus* sprouted earlier (the first week of December) than those of *C. vernus* (the end of January). In these species, leaf elongation precedes flowering by 8–10 weeks, whereas this difference ranged from 30 days before to 11 days after flowering in the case of the autumn crocuses (Figure 4).

A variance components analysis between and within species for the variable “days until flowering after planting” (Table S3) showed low intra-specific (4%) and high inter-specific variability (96%). Intra-specific differences in flowering time between the earliest and latest accessions were 5–20 days and up to 30 days for the most widely distributed populations of *C. serotinus* and *C. nevadensis* (Table S4). The populations within the same species subjected to different environmental conditions often differ as to phenotypic and genetic characters and plasticity [29,30]. Indeed, the widespread Spanish crocuses exhibit greater plasticity for the flowering time. In the inter-specific comparison, the biggest differences were recorded between autumn- and spring-flowering species.

In the natural habitats, corms become dormant at the beginning of the summer, when the temperatures rise. Thus, 563 accessions of 62 crocus species from the World Saffron and Crocus Collection (WSCC), grown in the same field, enter dormancy in mid-May, with no significant inter-specific differences [31]. This phenological behavior is typical for all Crocus species, which are adapted to the dry summers of the Mediterranean and the Irano-Turanian floristic regions [9]. In our experiments ex situ, the fruit ripening of every species coincided at the end of May with no differences among species, and the inter-specific variability in leaf senescence at the end of May was minor (Figure 4; Table S3).

### 3.3. Temperature Requirements for Flower Initiation

The time of flowering initiation within the renewal bud could cause inter-species differences in the flowering patterns. In order to elucidate the environmental effect on flower initiation in crocus species, we exposed dry corms to various storage temperatures. The floral transition of both the autumn and spring Spanish crocuses takes place in summer, after corm maturation and harvest, and 23 °C was found to be the optimal regime for flower initiation and differentiation in both autumn- and spring-flowering species (Table 3).

Altogether, 72–100% of the corms of four species initiated flowers when stored at this temperature. Low (5 °C and 10 °C) or high (30 °C and 35 °C) temperatures restricted or even damaged flower initiation. Only 15.4% of *C. nudiflorus* corms developed flower primordia at 10 °C, but malformations were observed; while 66.7% of the autumn-flowering *C. serotinus* corms stored at 30 °C initiated normal flowers, but this process failed completely when stored at 35 °C (Table 3).

Corm storage at 23 °C was also beneficial for flower initiation in saffron [16]. However, unlike most wild crocuses, cultivated saffron is able to develop flower primordia at 30 °C. *C. serotinus* also behaves in this way. This ability could be related to the wide distribution and greater ecological plasticity of *C. sativus* and *C. serotinus* and their adaption to the locations with higher summer temperatures.

**Figure 5:**
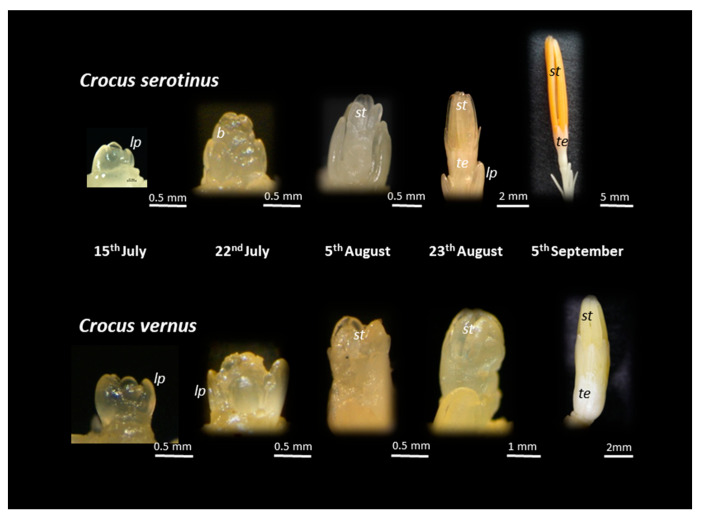
Microphotographs of flower differentiation in the autumn-flowering *C. serotinus* and spring-flowering *C. vernus*. After harvest, corms were stored at 23 °C until mid-September. On each date, at least 80% of the corms presented flower buds as shown in the figure. lp: leaf primordia; b: bract; st: stamen; te: tepal.

Floral differentiation is initiated in the apical meristem soon after the harvest of crocus corms, similar to other Mediterranean and Irano-Turanian geophytes, such as *Tulipa* [32], *Paeonia* [33], *Ornithogalum dubium* [34], or *Narcissus* [35]. It is interesting to note that flower initiation and differentiation show similar patterns in the autumn and spring crocuses until the beginning of August (Figure 5). By mid-July, a dome-shaped shoot apex with leaf primordia initiated at the flanks of the meristem was observed in all of the species. Bracts were initiated at the edges of the meristem at the end of July, and stamen initiation took place in the first days of August.

In August, the differentiation of the flowers of autumn species occurs at a much faster rate. By early September, the first whorl of tepals of *C. serotinus* are already differentiated and the stamens became yellow–orange and elongated to 2 cm (Figure 5). At the same time, the flower of *C. vernus* is still not differentiated, and the stamens remain white and short (0.7–0.8 cm). Therefore, in autumn, when temperatures fall, two species show variations in their floral differentiation and, when water is available, only the autumn species begin the aboveground cycle.

Our results indicate that both the spring and autumn crocuses belong to the florogenetic “Tulip type” of geophytes [1]. In this type, the reproductive development is induced by high temperatures without cold induction, and flower initiation takes place within the storage organ, during the “dormancy” period prior to growth [35,36]. The first steps towards understanding the molecular regulation of geophytes requiring elevated temperatures for the process have been taken [37,38,39,40], and regulatory networks of flower initiation and differentiation of Tulip, Narcissus and Crocus are currently under investigation. However, in view of our observations, the differences between the flowering schedules of the spring- and autumn-flowering crocuses should not be attributed to the flower initiation, but to the variations in the underground growth of the already-formed flowers and their elongation.

### 3.4. Effect of Growth Temperatures on Flowering

The corms of the autumn-flowering *C. serotinus* and spring-flowering *C. nevadensis* and *C. vernus* were stored at 23 °C from their harvesting in June until they were planted in September and exposed to various temperature regimes (Table 1). Exposure to 25 °C inhibited the sprouting of all three species, and no flowering was observed in these plants.

**Figure 6:**
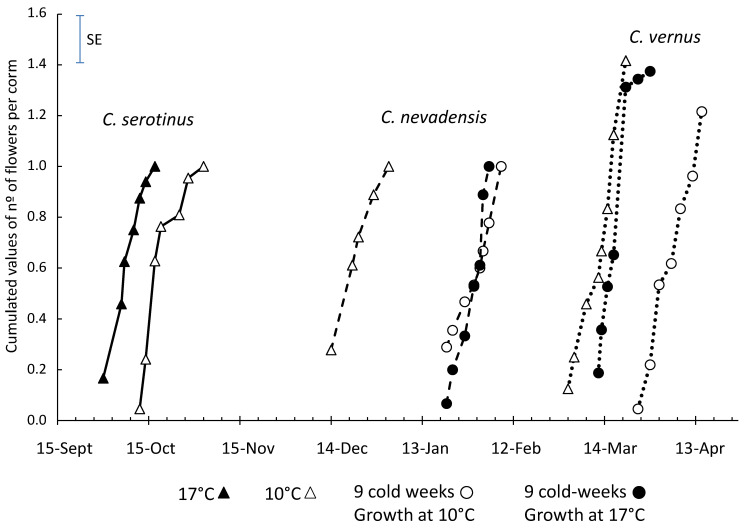
Cumulated values of number of flowers per corm in the autumn *C. serotinus* and the spring-flowering *C. nevadensis* and *C. vernus* exposed to various temperature regimes. *C. serotinus* was grown at a constant 10 °C or 17 °C. Corms of *C. nevadensis* and *C. vernus* were exposed to 9 cold weeks prior to growth at 10 or 17 °C. In addition, they were grown at a constant 10 °C. Each treatment contained two to four replicates of 10–30 corms (see Table 1 for a more detailed overview of treatments).

In the case of *C. serotinus*, growth at 17 °C promoted flowering when compared with colder conditions (Figure 6). When exposed to 17 °C, flowers appeared in a few days, and the whole population bloomed for 2 weeks, until the end of September. At 10 °C, growth was delayed by 4–5 weeks, and flowering was prolonged until the first 10 days of November.

Autumn crocuses with a Mediterranean life strategy [27] grow fast after the drop in temperature in early autumn. No accumulation of chilling units is required; merely the signal of a fall in temperature induces flower bud elongation. Interestingly, a higher growth temperature (17 °C vs. 10 °C) promoted anthesis, but shortened the flowering period and the flower life, and reduced the flower size (Table 4).

During the anthesis of *C. serotinus*, spring-flowering *C. nevadensis* and *C. vernus* still did not sprout, and only stamen differentiation and slow tepal development was recorded in the underground flower buds (Figure S3). From mid-October until December, the growth of the floral bud of *C. nevadensis* progressed faster than that of *C. vernus*, and flowering occurred in the second half of December and in early March, respectively (Figure 6).

Like most geophytes, crocuses are thermo-periodic. High temperatures of 25–30 °C induce flowering in the underground bud, but prevent sprouting; therefore, a drop in temperature is required to promote scape elongation and anthesis. A warm-cold-warm cycle is also required for *Allium*, *Tulipa*, *Narcissus*, *C. sativus,* and many other geophytes [16,36]. It has been shown that in spring ephemeral *C. vernus*, lower temperatures (12/8 °C vs. 18/14 °C or 10 °C vs. 17 °C) favor the final dry mass and cell size of daughter corms, while the leaves lasted longer at higher temperatures [28]. Earlier leaf senescence and the reduced growth of the corm when the temperature is increased from 10 to 17 °C is also reported for *C. vernus* by Lundmark et al. [41]. In our experiments, a higher growth temperature (17 vs. 10 °C) shortened the period of anthesis and reduced the flower size in both autumn- and spring-flowering species (Table 4).

The cold period prior to growth at 10 or 17 °C did not promote flowering and even delayed it by 40 days in the case of *C. nevadensis* and by 20 days in that of *C. vernus* (Figure 6). Rees [42] suggested that the spring crocuses have the Irano-Turanian type of annual cycle and require a prolonged cold period at 4–9 °C for dormancy release. Also, in the forcing protocols for North America [43] and Europe [12], it was stated that 13–15 weeks at 2–9 °C are recommended for the purposes of forcing commercial varieties of spring-flowering crocuses. Our results indicate that wild species from Spain do not fit this pattern and winter temperatures are not required for dormancy release and do not accelerate flowering. Quite the opposite: low temperatures significantly delay flowering in both studied species.

**Figure 7:**
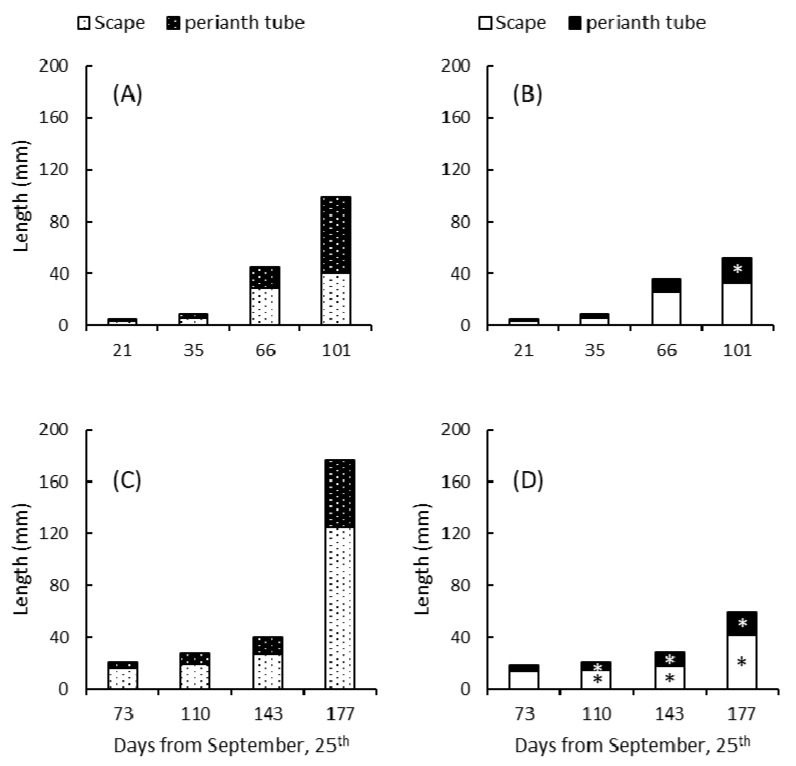
Variation in scape and perianth tube length in *C. nevadensis* (**A**,**B**) and *C. vernus* (**C**,**D**), when potted plants were grown at 10 °C (**A**,**C**) or exposed to a cooling period from 20 November 2010 to 20 January 2011 (**B**,**D**). * Significant differences in length as compared to those plants grown at 10 °C. Five plants per date and treatment were measured.

This fact is also supported by recorded differences in the growth rates of the scape and the perianth tube in the flower bud prior to emergence. In both spring-flowering species, lower temperatures of 2–5 °C inhibited the elongation of both reproductive structures during their underground development (Figure 7). The cataphylls remained closed after breaking through the soil surface, and anthesis occurred only when the temperature rose, 40–45 days later than in the corms grown at a constant 10 °C without cold treatment.

## 4. Conclusions

**Figure 8:**
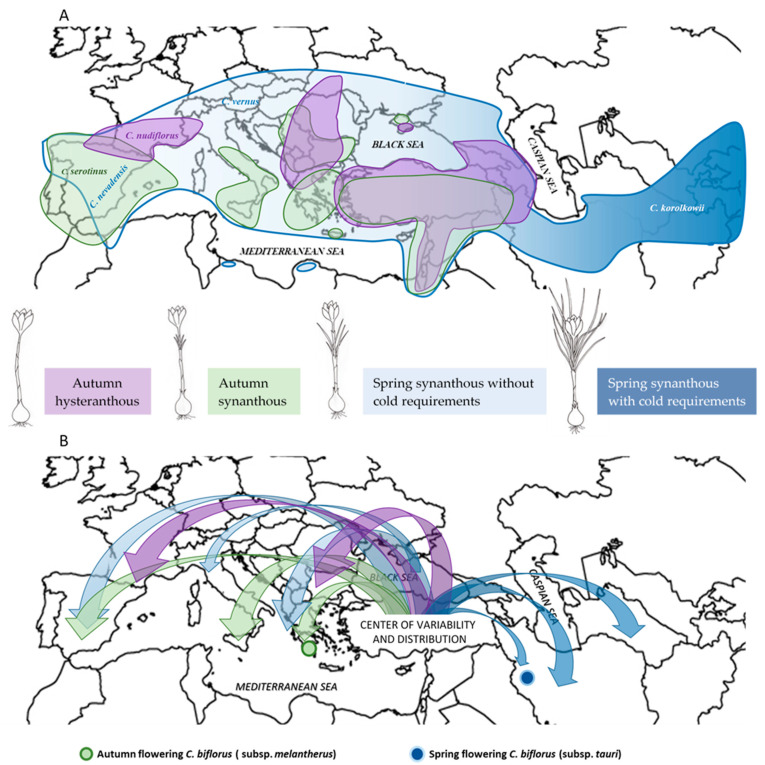
(**A**) Scheme of distribution of the different eco-morphological groups of 87 Crocus in the different regions of Eurasia. Some examples of Mediterranean (*C. serotinus*, *C. nudiflorus*; *C. nevadensis*, *C. vernus*) and Irano-Turanian species (*C. korolkowii*) have been specifically located on the map. (**B**) Possible evolutionary pathways of *Crocus* species from the center of variability and distribution in Turkey to Mediterranean regions and Central Asia. The advanced flowering in species spreading through the Mediterranean area is well represented in the species *C. biflorus*, with autumn flowering subspecies in Greece and spring flowering subspecies in Turkey and N.W. Iran (Data from Mathew, 1982 [9]; Rukšāns, 2010 [8]; Fernández et al., 2011 [19]).

The reproductive success of plants is affected by genetic and environmental factors and biotic interactions [44]. A great genetic variability of flowering phenology is harbored within genera and species and allows plant populations to evolve rapidly in response to local conditions, as evidenced by many examples [45,46]. One of the results of such an adaptation is histeranthy—the ability of plants to flower “off-season” and to distinguish between reproductive and vegetative periods in its annual cycle. In this context, the genus *Crocus* is extremely variable, not only as regards its morphological traits [47], but also in terms of the developmental patterns and environmental requirements. This genus represents a unique example of the “ongoing evolution” of hysteranthous species from synanthous relatives during their spread through the Mediterranean climates (Figure 8).

Spring synanthous species are represented by the Series Orientales, grown in Central Asia [9], the easternmost part of the genus’s distribution area. It is found in the Irano-Turanian region and requires a prolonged cold period for flowering [12,48]. These spring-flowering species show a synanthous phenological pattern.

Autumn hysteranthous species are distributed in the west of Central Asia, Transcaucasia and the northeast of Turkey, in the Balkan region, and reaching as far as the mountainous regions of northern Italy, southern France, and northern Spain. We propose that the reduction in the cold requirements and the hysteranthous annual cycle were acquired during the evolution of the genus and the plant’s distribution to the milder climate of Europe and the Mediterranean region (Figure 8). *Crocus* species from this group grow at a high altitude and flower in autumn, while leaves appear only in spring. Leaf elongation after autumn flowering would be useless in these species, because the winter cold and snow prevent photosynthesis.

Autumn synanthous and sub-hysteranthous species are present in the regions with a Mediterranean climate. Contrary to Central Asian crocuses, no Spanish *Crocus* species require cold for flowering. The prolonged summer dormancy period in this region is compensated by the vegetative period, photosynthesis, and reserve accumulation during autumn and winter. In autumn-flowering species, the leaves elongate shortly before, simultaneously with, or just after flowering. This life cycle is characteristic for the crocuses from the southwesternmost part of the distribution area. Only *C. nudiflorus*, which grows at a high altitude, shows advanced flowering and hysteranthia.

Turkey is the centre of diversity from which the genus *Crocus* could spread to the Mediterranean area and to the Irano-Turanian region. The shift towards the Mediterranean area made advanced autumn flowering possible. However, the Asian species are spring crocuses. An example of the variation in the flowering pattern with this shift is *C. biflorus*, with the autumn flowering subspecies in Greece (*C. biflorus* subsp. *melantherus*) and the spring flowering subspecies *C. biflorus* subsp. *tauri* in N.W. Iran (Figure 8B). Phylogenetic analysis based on molecular data [49] also suggests that similar adaptation strategies appear independently in distantly-related clades of *Crocus*. Thus, autumn crocus *C. cambessedessii* and spring crocus *C. vernus* belong to section *Crocus* (or subgen. *Involucrati*), whereas autumn crocus *C. speciosus* and spring crocuses *C. nevadensis* or *C. carpetanus* are members of section *Nudiscapus* (subgen. *Nudiflori*).

This inter-specific variation in the flowering phenology within the large genus *Crocus* represents a model for purposes of understanding the evolution of geophytes in general, and especially in the context of climate changes. Further research on molecular level would be necessary to enlighten ecological adaptations within the genus.

Our results suggest that the transition to advanced autumn flowering and hysteranthy is tightly connected with the genetic and environmental regulation of the elongation of the flower stalk and perianth. All of the studied crocus species initiate flowers in summer, but only in some of them do the flowers elongate in autumn, with no cold requirements. The molecular aspects of flowering transition and flower differentiation in saffron have already been reported [40], and several genes involved in this process have been isolated [50,51,52]. Further research into the molecular mechanisms of flower development will not only allow us a better understanding of the evolution of the genus, but also permit us to find marker genes for flowering time. Taxonomic and functional genomics should be future research goals.

## Figures and Tables

**Table 1:** Experimental layout of storage and growth conditions of three Crocus species. Corms were harvested in June, stored at constant 23 °C for 2 months and then exposed to various growth conditions. ↓ Progressive decrease in temperature. ↑ Progressive increase in temperature.

Species		Storage Temperature before Planting				Temperature Regime after Planting							
		Jun	Jul	Aug	Sep	Oct	Nov	Dec	Jan	Feb	Mar	Apr	May
1	Autumn Flowering *C. serotinus*					25 °C							
2		23 °C				17 °C							
3						↓	10 °C						
4	Spring Flowering *C. nevadensis* *C. vernus*					25 °C							
5						↓	10 °C						
6		23 °C				↓		5–2–5 °C		10 °C			
7						↓		5–2–5 °C		↑	17 °C		

**Table 2:** Flowering time and environmental data of 58 native populations of seven Spanish crocus species. Days from August 1st to flowering (DTF, Mean value) are calculated for the comparison with common garden experiments.

Species	Population Number	Flowering Period			Altitude (m.a.s.l)	Temperature during Flowering (°C)	
		Earliest	Latest	DTF		Max.	Min.
*C. serotinus*	14	17 Sep	28 Oct	64 a	320–1454	18	6
*C. nudiflorus*	13	12 Sep	15 Oct	59 a	910–2100	10	4
*C. clusii*	8	01 Nov	02 Nov	93 b	14–88	19	18
*C.cambessedesii*	4	07 Nov	09 Nov	99 b	30–250	20	18
*C. nevadensis*	12	16 Feb	04 Mar	210 c	600–1800	11	4
*C. carpetanus*	5	24 Feb	14 Mar	211 c	1100–1468	8	6
*C. vernus*	2	25 Apr	03 May	271 d	1560	10	9

Different letters indicate significant differences (*p* < 0.05) among species.

**Table 3:** Effect of the corm storage temperature on the flower primordia formation in the autumn- and spring-flowering crocus species. Corms were stored from mid-June to mid-September. Percentages of corms with flower primordia (microscopically observed) corresponding to the average values of two experiments carried out in consecutive years are presented. Thirty-five to 45 corms per treatment and species were observed each year.

Storage Temperature (°C)	Percentage of Flower Initiation							
	*C. serotinus*		*C. nudiflorus*		*C. nevadensis*		*C. vernus*	
5	-		0	c	-		-	
10	0	c	15.4	b	0 *	b	0 *	c
23	100	a	75	a	77.8	a	72.7	a
30	66.7	b	0 *	c	0	b	7.6	b
35	0	c	-		-		-	

* A very low percentage of corms with an abnormal development of floral meristems. Different letters indicate significant differences (*p* < 0.05) among treatments for each species.

**Table 4:** Effect of temperature on the flower size, flowering period and the average life of the flower of the crocus species. The corms were stored at 23 °C from their harvest at the end of June, until planting in mid-September. Flower length is measured during anthesis. Flower period is recorded as the length of time a plant population is flowering. Flower life is measured as the days from when one individual flower emerges until it begins to wilt. Fifteen plants per treatment were measured.

Species	Growth Temperatures	Flowering Period (days)		Flower Length (mm)		Flower Life (days)	
*C. serotinus*	10 °C	17	a	223	b	8	b
	17 °C	9	b	204	a	6	a
*C. nevadensis*	10 °C	18	b	88	a	8	b
	Cold + 10 °C	7	a	101	b	8	b
	Cold + 17 °C	4	a	85	a	5	a
*C. vernus*	10 °C	16	b	97	a	10	b
	Cold + 10 °C	16	b	108	b	9	b
	Cold + 17 °C	7	a	96	a	7	a

Different letters indicate significant differences (*p* < 0.05) among treatments for each species.

## Data Availability

All related data are available within the manuscript and its additional files. Additional information about the Crocus accessions is openly available in http://crocusbank.uclm.es (accessed on 26 October 2020).

## Supplementary Materials

The following are available online at https://www.mdpi.com/2223-7747/10/3/477/s1, Table S1. Location data of Crocus accessions evaluated in their natural environment, Table S2. Accessions evaluated in the study of the phenology of Spanish *Crocus* species under cultivation. Accessions and Spanish provinces where they were collected are indicated for each species, Table S3. Variance components in phenological traits of Spanish *Crocus* species, Table S4. Flowering time of the earliest and latest populations of 112 accessions of seven Spanish crocuses under the same environmental conditions (common garden experiment), Figure S1. Variations in temperature throughout the year in the experimental field of the CIAF during the trial whose aim was to study the phenology of Spanish *Crocus* species grown under cover, Figure S2. Correlation between altitude of natural habitats of autumn-flowering crocuses and their flowering. Beginning of flowering calculated as days after 1 August 2011. Figure S3. Sequence of flower development under (yellow frame) and aboveground (green frame), and senescence (brown frame) for autumn *C. serotinus* (**A**) and spring *C. nevadensis* and *C. vernus* (**B**,**C**). After harvesting, corms were stored at 23 °C and planted in September, after flower bud differentiation. The temperature decreased to 10 °C in early October. Phenological stages were represented by at least 80% of the plants.
