# Supplementary material for: Ongoing Evolution in the Genus Crocus: Diversity of Flowering Strategies on the Way to Hysteranthy
Source: Plants (Basel). 2021 Mar 3;10(3):477. doi: 10.3390/plants10030477 (PMC7999489; doi:10.3390/plants10030477)
Supplement: Supplementary file 1 [file plants-10-00477-s001.zip › Figure S1.pdf]

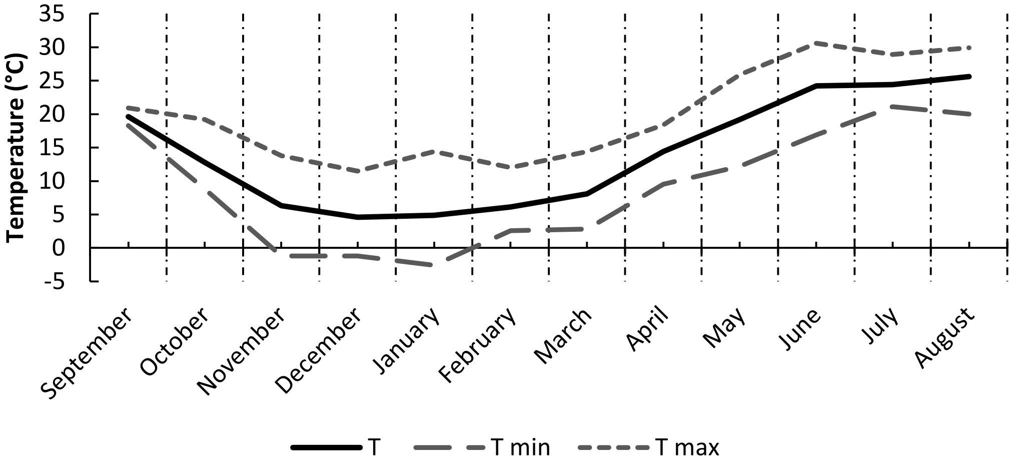

Figure S1. Variations in temperature throughout the year in the experimental field of the CIAF during the trial whose aim was to study the phenology of Spanish Crocus species grown under cover.
