# Supplementary material for: Ongoing Evolution in the Genus Crocus: Diversity of Flowering Strategies on the Way to Hysteranthy
Source: Plants (Basel). 2021 Mar 3;10(3):477. doi: 10.3390/plants10030477 (PMC7999489; doi:10.3390/plants10030477)

Figure S2. Relation between altitude at which autumn-crocus grow in their natural habitats and the flowering time.

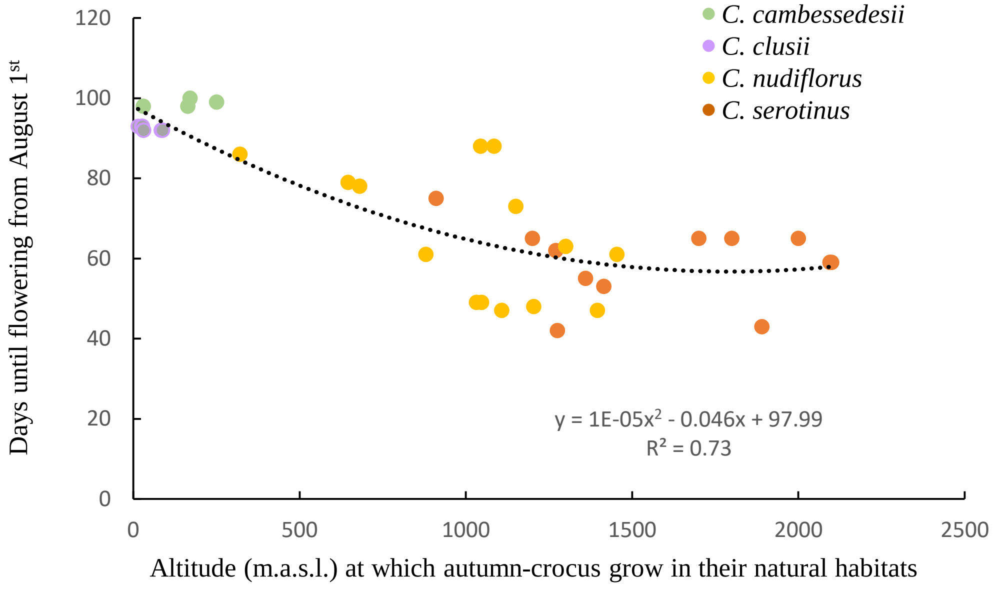

Supplement: Supplementary file 1 [file plants-10-00477-s001.zip › Figure S2.pdf]
