# Supplementary material for: Ongoing Evolution in the Genus Crocus: Diversity of Flowering Strategies on the Way to Hysteranthy
Source: Plants (Basel). 2021 Mar 3;10(3):477. doi: 10.3390/plants10030477 (PMC7999489; doi:10.3390/plants10030477)

Figure S3. Sequence of flower development under (yellow frame) and aboveground (green frame), and senescence (brown frame), for autumn *C. serotinus* (A) and spring *C. nevadensis* and *C. vernus* (B-C). After harvesting, corms were stored at 23°C and planted in September, after flower bud differentiation. The temperature decreased to 10°C in early October. Phenological stages represented by at least 80% of the plants. Cataphylls were removed in yellow framed photos.

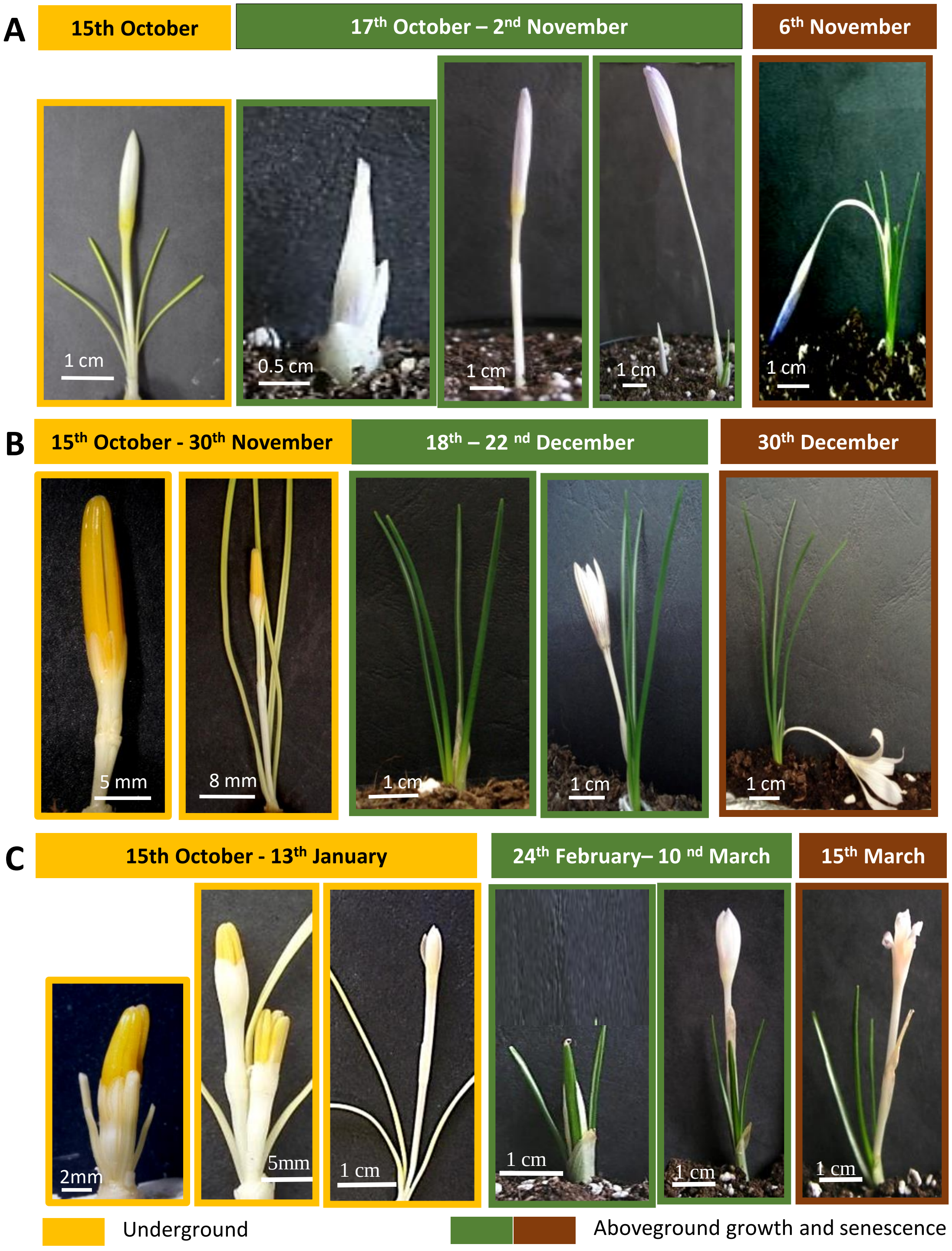

Supplement: Supplementary file 1 [file plants-10-00477-s001.zip › Figure S3.pdf]
