# Supplementary material for: Ongoing Evolution in the Genus Crocus: Diversity of Flowering Strategies on the Way to Hysteranthy
Source: Plants (Basel). 2021 Mar 3;10(3):477. doi: 10.3390/plants10030477 (PMC7999489; doi:10.3390/plants10030477)
Supplement: Supplementary file 1 [file plants-10-00477-s001.zip › Table S1.pdf]

Table S1. Location data of *Crocus* accessions evaluated in their natural environment.

Accession number	Species	State	Province	Locality	Latitude	Longitude	Elevation (m.a.s.l.)
BCU001680	*C. cambessedesii* J. Gay	Islas Baleares	Mallorca	Es Repla (Arta)	39° 43' 33.8" N	3° 18' 31.6" E	164
BCU001682	*C. cambessedesii* J. Gay	Islas Baleares	Mallorca	Partida de Fuster (Capdepera)	39° 42' 32.3" N	3° 26' 36.1" E	30
BCU001683	*C. cambessedesii* J. Gay	Islas Baleares	Mallorca	Tunel de Formentor	39° 54' 46.2" N	3° 04' 16.1" E	170
BCU001685	*C. cambessedesii* J. Gay	Islas Baleares	Mallorca	Cami de Can Mameta (Caimari)	39° 46' 57.3" N	2° 53' 58.2" E	250
BCU002971	*C. carpetanus* Boiss. & Reut.	Comunidad de Madrid	Madrid	Montejo de la Sierra (Hayedo)	41° 04' 16.4" N	3° 31' 38.2" O	1242
BCU002972	*C. carpetanus* Boiss. & Reut.	Comunidad de Madrid	Madrid	Robregordo	41° 06' 52.8" N	3° 35' 38.3" O	1356
BCU002973	*C. carpetanus* Boiss. & Reut.	Comunidad de Madrid	Madrid	Robregordo	41° 07' 34.2" N	3° 36' 12.2" O	1468
BCU002974	*C. carpetanus* Boiss. & Reut.	Comunidad de Madrid	Madrid	Robregordo	41° 07' 11.9" N	3° 36' 11.7" O	1446
BCU002696	*C. carpetanus* Boiss. & Reut.	Galicia	Ourense	Muinos (Requias, Serra do Xures)	41° 53' 16" N	7° 56' 37" O	1100
BCU002969	*C. nevadensis* Amo	Castilla-La Mancha	Cuenca	Olmeda del Rey	39° 45' 52.4" N	2° 01' 36.91" O	1100
BCU002976	*C. nevadensis* Amo	Andalucía	Granada	Güejar Sierra	37° 07' 47" N	3° 26' 57.7" O	1610
BCU002977	*C. nevadensis* Amo	Andalucía	Granada	Monachil	37° 06' 53.4" N	3° 27' 02.5" O	1800
BCU002979	*C. nevadensis* Amo	Andalucía	Granada	Monachil	37° 07' 37.6" N	3° 27' 05.5" O	1688
BCU002980	*C. nevadensis* Amo	Andalucía	Jaén	Quesada	37° 52' 04.08" N	3° 00" 58.7" O	1172
BCU002981	*C. nevadensis* Amo	Andalucía	Jaén	Cazorla	37° 53' 50.7" N	2° 59' 17.8" O	1145
BCU002982	*C. nevadensis* Amo	Andalucía	Jaén	Quesada	37° 52' 18.3" N	3° 00' 15.9" O	1333
BCU002356	*C. nevadensis* Amo	Castilla-La Mancha	Cuenca	Motilla del Palancar	39° 31' 48.84"N	1° 54' 45.83" O	822
BCU002693	*C. nevadensis* Amo	Castilla-La Mancha	Cuenca	Altomira	40° 10' 52.47" N	2° 49' 34.14" O	1150
BCU002695	*C. nevadensis* Amo	Castilla-La Mancha	Cuenca	Olmeda del Rey	39° 45' 52.4" N	2° 01' 36.91" O	1100
BCU002697	*C. nevadensis* Amo	Navarra	Pamplona	Lazagurria	42° 29' 23.2" N	2° 12' 59.9" O	700
BCU002698	*C. nevadensis* Amo	Navarra	Pamplona	Tafalla	42° 34' 47.1' N	1° 42' 09.7" O	600
BCU003123	*C. nudiflorus* S.M.	La Rioja	La Rioja	Villoslada de cameros	42° 02' 49.47" N	2° 40' 44.13" O	1415
BCU002556	*C. nudiflorus* S.M.	Aragón	Huesca	Benasque	42° 40' 0.93" N	0° 3' 7.65" E	1800
BCU002557	*C. nudiflorus* S.M.	Aragón	Huesca	Benasque	42° 40' 6.89" N	0° 3' 9" E	2000

Accession number	Species	State	Province	Locality	Latitude	Longitude	Elevation (m.a.s.l.)
BCU002558	*C. nudiflorus* S.M.	Aragón	Huesca	Benasque	42° 40' 87" N	0° 3' 6.26" E	1700
BCU002559	*C. nudiflorus* S.M.	Aragón	Huesca	Benasque	42° 36' 87" N	0° 3' 1.85" E	1200
BCU002735	*C. nudiflorus* S.M.	Aragón	Huesca	Laspuña	42° 28' 56.4" N	0° 14' 29.2" E	1275
BCU002736	*C. nudiflorus* S.M.	Aragón	Huesca	Laspuña, Bassa de Mora	42° 33' 00.5" N	0° 19' 20.4" ' E	1890
BCU002737	*C. nudiflorus* S.M.	Cataluña	Lerida	Valle de Boi	42° 29' 12.95" N	0° 52' 26.40" E	2100
BCU002738	*C. nudiflorus* S.M.	Cataluña	Lerida	Valle de Boi	42° 29' 9.07" N	0° 52' 23.68" E	2095
BCU002739	*C. nudiflorus* S.M.	Cataluña	Lerida	Valle de Boi	42° 28' 58.56" N	0° 52' 23.21" E	2095
BCU002776	*C. nudiflorus* S.M.	La Rioja	La Rioja	San Millán de la Cogola	42° 14' 52" N	2° 55' 59" E	1360
BCU002777	*C. nudiflorus* S.M.	La Rioja	La Rioja	Manzanares de Rioja	42° 20' 49" N	2° 54' 57" E	1270
BCU002779	*C. nudiflorus* S.M.	La Rioja	La Rioja	Manzanares de Rioja	42° 22' 46" N	2° 54' 10" E	910
BCU002960	*C. clusii* J. Gay	Andalucía	Huelva	Doñana	37° 10' 21" N	6° 38' 57" O	30
BCU002961	*C. clusii* J. Gay	Andalucía	Huelva	Doñana	37° 10' 21" N	6° 38' 18" O	31
BCU002962	*C. clusii* J. Gay	Andalucía	Huelva	Hinojos	37° 16' 11" N	6° 25' 32" O	88
BCU002963	*C. clusii* J. Gay	Andalucía	Huelva	Hinojos	37° 16' 52" N	6° 23' 57" O	84
BCU002964	*C. clusii* J. Gay	Andalucía	Cádiz	Chipiona	36° 42' 45" N	6° 25' 4" O	14
BCU002965	*C. clusii* J. Gay	Andalucía	Cádiz	Puerto Real	36° 31' 22" N	6° 9' 5" O	27
BCU002966	*C. clusii* J. Gay	Andalucía	Cádiz	Laguna de la Paja	36° 23' 25" N	6° 7' 20" O	27
BCU002967	*C. clusii* J. Gay	Andalucía	Cádiz	Laguna de la Paja	36° 23' 26" N	6° 7' 50" O	25
BCU002959	*C. serotinus* Salisb.	Andalucía	Huelva	Aracena	37° 53' 0.35" O	6° 46' 38.6" O	880
BCU003172	*C. serotinus* Salisb.	Castilla y León	León	Villamanín	42° 55’ 57.3”N	5° 39’ 19.8” O	1204
BCU003173	*C. serotinus* Salisb.	Castilla y León	León	Barrios de Luna	42° 52’ 21.3”N	5° 55’ 7.9” O	1395
BCU003175	*C. serotinus* Salisb.	Castilla y León	Burgos	Las Cañadillas	42° 38’ 49.9”N	3° 40’ 27.4’’ O	1032
BCU003177	*C. serotinus* Salisb.	Castilla y León	Burgos	La Revuelta	42° 38’ 17.7” N	3° 40’ 57.9’’ O	1047
BCU003179	*C. serotinus* Salisb.	Castilla y León	León	Barrios de Luna	42° 49’ 38.2”N	5° 50’ 22” O	1108
BCU001704	*C. serotinus* Salisb.	Castilla-La Mancha	Toledo	Villarrubia de Santiago	40° 00' 24" N	3° 22' 33" O	680
BCU002618	*C. serotinus* Salisb.	Comunidad Valenciana	Valencia	Caroche (Vértice Geo)	39° 05' 25" N	0° 54' 56" O	1085

Accession number	Species	State	Province	Locality	Latitude	Longitude	Elevation (m.a.s.l.)
BCU002619	*C. serotinus* Salisb.	Comunidad Valenciana	Valencia	Caroche (Balsa)	39° 05' 37" N	0° 55' 05" O	1044
BCU002685	*C. serotinus* Salisb.	Castilla-La Mancha	Ciudad Real	Almagro	38°53'34.04"	O3°44'9.67"	646
BCU002740	*C. serotinus* Salisb.	Castilla-La Mancha	Albacete	Tus, Sierra de Segura	38° 24' 09.1" N	2° 25' 09.4" O	1300
BCU002741	*C. serotinus* Salisb.	Galicia	A Coruña	Mazaricos	42° 54' 33.26"N	9° 06' 16.97" O	320
BCU002742	*C. serotinus* Salisb.	Castilla-La Mancha	Albacete	Tus, Sierra de Segura	38° 24' 01.3" N	2° 24' 07.9" O	1454
BCU002743	*C. serotinus* Salisb.	Castilla-La Mancha	Cuenca	Altomira	40° 10' 52.47" N	2° 49' 34.14" O	1150
BCU002392	*C. vernus* (L.) Hill	Aragón	Huesca	Jaca (Refugio de Boyeros)	42° 44' 25" N	0° 35' 11" O	1560
BCU003198	*C. vernus* (L.) Hill	Aragón	Huesca	Jaca (Refugio de Boyeros)	42° 45' 49" N	0° 37' 23" O	1581
