# Supplementary material for: Ongoing Evolution in the Genus Crocus: Diversity of Flowering Strategies on the Way to Hysteranthy
Source: Plants (Basel). 2021 Mar 3;10(3):477. doi: 10.3390/plants10030477 (PMC7999489; doi:10.3390/plants10030477)
Supplement: Supplementary file 1 [file plants-10-00477-s001.zip › Table S2.pdf]

Table S2. Accessions evaluated in the study of the phenology of Spanish *Crocus* species under cultivation. Accessions and Spanish provinces where they were collected are indicated for each species.

SPECIES	PROVINCE	ACCESSIONS
C. cambessedesii	Mallorca	BCU001680, BCU001682, BCU001683, BCU001685
	Nursery	BCU003130
C. carpetanus	Ávila	BCU002835, BCU002836, BCU002837, BCU002838
	Madrid	BCU002971, BCU002972, BCU002973, BCU002974, BCU002987
	Ourense	BCU002696
C. clusii	Huelva	BCU002960, BCU002961, BCU002962, BCU002963
	Cádiz	BCU002964, BCU002965, BCU002966, BCU002967
C. nevadensis	Cuenca	BCU002871, BCU002969, BCU003012, BCU002355, BCU002356, BCU002359, BCU002687, BCU002689, BCU002690, BCU002693, BCU002694, BCU002695
	Granada	BCU002976, BCU002977, BCU002979, BCU002980, BCU002386, BCU002387, BCU002388
	Huesca	BCU002992, BCU002993, BCU002994, BCU002999
	Jaén	BCU002981, BCU002982, BCU002380, BCU002381, BCU002382, BCU002383, BCU002384, BCU002385
	Lérida	BCU002988, BCU002989, BCU002990
	Pamplona	BCU002697, BCU002698
	Valencia	BCU001742
C. nudiflorus	Burgos	BCU003176
	Huesca	BCU002944, BCU003000, BCU003001, BCU003002, BCU003003, BCU003004, BCU003015, BCU002556, BCU002557, BCU002558, BCU002559
	La Rioja	BCU003123, BCU002776, BCU002779
	Lérida	BCU003005, BCU003006, BCU003007
C. serotinus	La Coruña	BCU002741
	Albacete	BCU002550, BCU002740, BCU002742
	Burgos	BCU003175
	Ciudad Real	BCU002366, BCU002367, BCU002685, BCU002686
	Cuenca	BCU002873, BCU002970, BCU003013, BCU002743, BCU002775
	Granada	BCU002978
	Huelva	BCU002959
	Jaén	BCU001655, BCU001657
	León	BCU003172, BCU003173, BCU003174, BCU003177, BCU003178, BCU003179, BCU003180, BCU003181
	Toledo	BCU001704, BCU001705
	Valencia	BCU002618, BCU002619
C. vernus* subs. *albiflorus	Nursery	BCU002998, BCU003018, BCU001822, BCU002730
