# Supplementary material for: Ongoing Evolution in the Genus Crocus: Diversity of Flowering Strategies on the Way to Hysteranthy
Source: Plants (Basel). 2021 Mar 3;10(3):477. doi: 10.3390/plants10030477 (PMC7999489; doi:10.3390/plants10030477)
Supplement: Supplementary file 1 [file plants-10-00477-s001.zip › Table S3.pdf]

Table S3. Variance components in phenological traits of Spanish *Crocus* species.

Phenological traits*	Variance partitioning			
	Between species		Within species	
	%	*fd*	*%*	*fd*
Sprouting time	83.01	6	16.99	80
Leaf emergence	69.40	6	30.60	97
Flowering time	96.40	6	3.60	54
Leaf senescence	43.68	6	56.32	100

* Measured as the number of days between the date of planting (1st of August) and a certain stage.
