# Supplementary material for: Ongoing Evolution in the Genus Crocus: Diversity of Flowering Strategies on the Way to Hysteranthy
Source: Plants (Basel). 2021 Mar 3;10(3):477. doi: 10.3390/plants10030477 (PMC7999489; doi:10.3390/plants10030477)
Supplement: Supplementary file 1 [file plants-10-00477-s001.zip › Table S4.pdf]

**Table S4**. Flowering time of the earliest and latest populations of 112 accessions of seven Spanish crocuses under the same environmental conditions (common garden experiment).

| **Species** | **Time of flowering** | |
|---|---|---|
| | Earliest population | Latest population |
| *C. serotinus* | *5-Oct* | *4-Nov* |
| *C. clusii* | *11-Nov* | *16-Nov* |
| *C. nudiflorus* | *21-Oct* | *7-Nov* |
| *C. cambessedesii* | *28-Oct* | *9-Nov* |
| *C. nevadensis* | *10-Jan* | *1-Mar* |
| *C. carpetanus* | *23-Jan* | *6-Feb* |
| *C. vernus* | *6-Mar* | *28-Mar* |
